# Droplet-Transmitted Infection Risk Ranking Based on Close Proximity Interaction

**DOI:** 10.3389/fnbot.2019.00113

**Published:** 2020-01-21

**Authors:** Shihui Guo, Jubo Yu, Xinyu Shi, Hongran Wang, Feibin Xie, Xing Gao, Min Jiang

**Affiliations:** ^1^School of Informatics, Xiamen University, Xiamen, China; ^2^Department of Orthopaedic Trauma, Zhongshan Hospital, Xiamen University, Xiamen, China

**Keywords:** influenza-like infection, person re-identification, multi-person pose estimation, infection risk ranking, multi-tasking

## Abstract

We propose an automatic method to identify people who are potentially-infected by droplet-transmitted diseases. This high-risk group of infection was previously identified by conducting large-scale visits/interviews, or manually screening among tons of recorded surveillance videos. Both are time-intensive and most likely to delay the control of communicable diseases like influenza. In this paper, we address this challenge by solving a multi-tasking problem from the captured surveillance videos. This multi-tasking framework aims to model the principle of Close Proximity Interaction and thus infer the infection risk of individuals. The complete workflow includes three essential sub-tasks: (1) person re-identification (REID), to identify the diagnosed patient and infected individuals across different cameras, (2) depth estimation, to provide a spatial knowledge of the captured environment, (3) pose estimation, to evaluate the distance between the diagnosed and potentially-infected subjects. Our method significantly reduces the time and labor costs. We demonstrate the advantages of high accuracy and efficiency of our method. Our method is expected to be effective in accelerating the process of identifying the potentially infected group and ultimately contribute to the well-being of public health.

## 1. Introduction

The most frequent infectious diseases in humans—and those with the highest potential for rapid pandemic spread—are usually transmitted via droplets during close proximity interactions (Salathé et al., 2010). Such infectious diseases include influenza, common colds, whooping cough, SARS-CoV, and many others. Influenza alone leads to a projected annual cost of 2.0-5.8 billion USD for the American health-care system (Yan et al., 2017). It is critical to identify the group of individuals who are in close contact with the diagnosed patient, in order to understand and mitigate the spread of the aforementioned pandemic diseases.

Previous attempts model the contact networks relevant for disease transmission by using online questionnaire (Ibuka et al., 2016), surveys (Leung et al., 2017), and wearable devices (Smieszek et al., 2016; Ozella et al., 2018). Manual approaches (surveys and interviews) require a significant amount of human efforts, while wearable devices introduce additional cost and are limited to small-scale study. Open challenges remain in the development of methods to fast capture the contact networks. Given the high density of surveillance cameras in metropolitans, the impact of using captured videos to identify the contact networks is under-explored. However, two significant challenges exist for this vision-based method: (1) re-identify the diagnosed patient in non-overlapping monitor cameras and (2) assess the potential risk of infection in the exposed population. The most popular solution to identify a specific person from videos is currently face recognition. However, poor illumination and camera viewpoint make it difficult for existing face recognition method to achieve satisfactory performance. Overlapping and occlusion of multiple faces also create significant difficulties. Meanwhile, it is non-trivial to assess the infectious risk from the captured video quantitatively. How to obtain a robust estimation of the interaction between the detected subjects in the video is still an open question.

We propose a novel framework to automatically evaluate the infection risk based on the principle of *Close Proximity Interaction*. Our success leverages the advantages of Artificial Intelligence (AI) systems over human beings in solving multiple tasks simultaneously. The accurate identification of this potentially-infected group can only be achieved with an integrative understanding of personal identity, spatial and temporal contexts from the video sequences. Such a wide range of information is processed by individual sub-tasks, including person detection, re-identification, depth and pose estimation. The user study shows that our method is effective in reducing the time and labor costs, and produces consistent results as human screening.

To this end, we made the following contributions:

- We propose a novel framework to evaluate the infection risk of identified individuals. This framework is constructed upon multi-tasking capabilities of modern techniques of computer vision. Our method effectively addresses the problem of infectious disease prevention, greatly reducing labor and time costs.
- We quantitatively model the principle of *Close Proximity Interaction* for assessing and ranking the infection risk. This is achieved by robustly reconstructing the 3D joint trajectories, based on 3D depth and pose estimation. The proposed metric takes distance as well as mutual contact between subjects into account.
- We evaluate our method in real-world environments including indoor office, and other scenarios with massive human traffic (e.g., shopping mall, hospital, public transport). The results show that our automatic method is not only time-efficient but also produces consistent prediction results as human observers.

The rest of this paper is structured as follows. Section 2 summarizes the related works, and section 3 describes the proposed framework to model the principle of *Close Proximity Interaction*, including the cornerstones to build this framework. Section 4 presents the results from our experiments, and section 5 discusses the failure cases and limitations of our method. Section 7 concludes this work and points out the directions for future efforts.

## 2. Related Work

### 2.1. Infectious Disease Monitor

Monitoring the spread of infectious disease is critical for taking prompt actions to control the expansion. The contact in close distance between an infectious individual and the population leads to the spread of respiratory infections (Leung et al., 2017). This paper investigates the diseases transmitted via droplets.

The conventional methods started with social surveys, by asking participants to report their contact patterns, including the number/duration of contacts and other demographical information (including age, gender, household size) (Eames et al., 2012; Read et al., 2014; Dodd et al., 2015). Understanding the contact pattern allows us to build parameterized models and capture the transmission patterns. Leung et al. (2017) proposed a diary-based design, using both paper and online questionnaires, and found out that the approach of using paper questionnaires leads to an increasing report of contacts and longer contact duration than using online questionnaires. However, conducting such social surveys and questionnaires requires a significant amount of time and effort.

Researchers use wearable devices to analyze the contact patterns among a group of individuals. A recent work measured face-to-face proximity between family members within 16 households with infants younger than six months for 2-5 consecutive days of data collection (Ozella et al., 2018). Researchers compared the two methods of reporting with paper diaries and recording with wearable sensors, to monitor the contact pattern at a conference (Smieszek et al., 2016). They found out that reporting was notably incomplete for contacts <5 min, and participants appear to have overestimated the duration of their contacts. The typical device is RFID-based and proves to be useful in a variety of scenarios, including a pediatric hospital (Isella et al., 2011), a tertiary care hospital (Voirin et al., 2015), and a primary school (Stehlé et al., 2011). The merit of using wearable devices is a high-resolution measurement of contact matrices between individuals with the device. However, it is not feasible to apply to a wide, dynamic, and unconstrained scenario.

Different from existing methods, our work utilizes the surveillance cameras as the capture device and process the video input with the state-of-the-art techniques in computer vision. Our method quantitatively modeled the principle of close proximity interaction and introduced a graph structure to represent the contact pattern.

### 2.2. Person Re-identification

Person re-identification is a long-standing and significant problem that has profound application value for a wide range of fields such as security, health care, business. It aims at re-identifying the person of interest from a collection of images or videos taken by multiple non-overlapping cameras in a large distributed space over a prolonged period. Re-ID is fundamentally challenging due to three difficulties: (1) diverse visual appearance changes caused by variations in view angle, lighting, background clutter, and occlusion. (2) difficulties in producing discriminating feature representation invariant to background clutter. (3) over-fitting problem due to the limited scale of a tagged dataset.

Two types of solutions are proposed to address these problems. One is to learn a more distinctive feature representation to make a trade-off between recognition accuracy and generalization ability. The other is to leverage the Siamese neural network and triplet loss to minimize the loss of images with the same identity and maximize that with different identities. We briefly survey the person re-identification literature from these two aspects in this paper.

#### 2.2.1. Improvements in Feature Representation

Improvements in feature representation mainly achieved by leveraging local parts of the person. Representative methods applied part-informed features such as segmentation mask, pose, gait, etc. Pose sensitive model proposed by Saquib Sarfraz et al. (2018) incorporates both fine- and coarse-grained pose information into CNN to learn the feature representation without explicitly modeling body parts. The combined representation includes both the view captured by the camera and joint locations, which ensures the discriminating embedding. Song et al. (2018) proposed a mask-guided contrastive attention model to learn features separately from the background and human bodies. Their work takes the binary body mask as input to remove the background in pixel-level and use gait information as features. However, failure cases will happen when discriminative body parts are missing. Horizontal Pyramid Matching (HPM) approach is proposed by Fu et al. (2018), solving this problem by using partial feature representations at different horizontal pyramid scales and adopting average and max pooling for inter-person variations. For similarity measurement, metric learning approaches are exploited such as cross-view quadratic discriminant analysis (Liao et al., 2015), relative distance comparison optimization (PRDC algorithm) (Zheng et al., 2011), locally-adaptive decision functions (LADF) (Li et al., 2013) and etc.

#### 2.2.2. Siamese Neural Network Architecture

Siamese neural network architecture is also adopted to tackle the problem of person re-identification by taking image pairs or triplets (Ding et al., 2015) as input. Siamese CNN (S-CNN) for person re-identification was presented in Yi et al. (2014) and Li et al. (2014). Improvements such as Gated Siamese CNN (Varior et al., 2016) aimed at acquiring finer local patterns for discriminative capacity enhancement. Cheng et al. (2016) proposed a Multi-Channel Parts-Based CNN with improved triplet loss consisting of multiple channels to jointly learn the global full-body and local body-parts features. Triplet loss is also widely used to learn fine-grained similarity image metrics (Wang et al., 2014). Quadruplet loss Chen et al. (2017c) strengthens the generalization capability and leads the model to output with a larger inter-class variation and a smaller intra-class variation superior to triplet loss.

### 2.3. Multi-Person Pose Estimation

Multi-person pose estimation aims at recognizing and locating key points on multiple persons in the image, which is the basis for resolving the technical challenges such as human action recognition (HAR) and motion analysis. Single person pose estimation is based on the assumption that the person dominates the image content. Deep learning methods perform well when the assumption is satisfied. However, for our specific problem in this paper, the case of a single person in one captured image seldom happens. Thus, we focus on the survey of multiple people pose estimation problem here. Cases such as occluded or invisible key points and background clutter lead to significant difficulties for multi-person pose estimation. State-of-the-art approaches built on CNN can be mainly divided into two categories: bottom-up approaches and top-down approaches.

#### 2.3.1. Bottom-Up Approaches

Bottom-up approaches (Insafutdinov et al., 2016; Pishchulin et al., 2016; Cao et al., 2017) mainly adopt the strategy of detecting all key points in the image first and then matching poses to individuals. Deepcut (Pishchulin et al., 2016) casts the problem in the form of an Integer Linear Program (ILP), and the proposed partitioning and labeling formulation jointly solve the task of detection and pose estimation. A follow-up work, Deepercut (Insafutdinov et al., 2016), achieves better success by adopting image-conditioned pairwise terms with deeper ResNet (He et al., 2016). An open-source effort, Openpose (Cao et al., 2017), uses a non-parametric representation referred to as Part Affinity Fields (PAFs) for associating body parts with individuals, achieving real-time performance with high accuracy.

#### 2.3.2. Top-Down Approaches

Top-down approaches (Fang et al., 2017; Huang et al., 2017; Papandreou et al., 2017; Chen et al., 2018) are opposed to the former, locating and partitioning all persons in the image followed by utilizing single person pose estimation caches individually for each person. Cascaded Pyramid Network (CPN) (Chen et al., 2018) takes two steps to cope with overlapping or obscured keypoints: GlobalNet for easy recognized keypoints and RefineNet for hard one. Papandreou et al. (2017) leverages the Faster RCNN (Ren et al., 2015) as the person detector and the fully convolutional ResNet to predict heatmaps and offsets. The recent work based on Mask-RCNN (He et al., 2017) extends Faster RCNN to predict human keypoints by combining the human bounding box and the corresponding feature map.

### 2.4. Multi-Tasking Intelligence

Multi-tasking refers to the capability of solving many tasks simultaneously. The current advances of artificial intelligence outperform human beings in effortlessly handling multiple tasks without switching costs. There are a couple of mainstream techniques for solving multi-tasking problems.

One of the popular techniques is to use the evolutionary algorithm to tackle the problem of multi-tasking. This is referred to as evolutionary multi-tasking optimization. In classic EAs, different optimization problems are typically solved independently. Researchers proposed a variety of techniques, such as multi-factorial memetic algorithm (Chen et al., 2017a), opposition-based learning (Yu et al., 2019), cross-task search direction (Yin et al., 2019), explicit autoencoding (Feng et al., 2018), and cooperative co-evolutionary memetic algorithm (Chen et al., 2017b), for the purpose of solving the multi-tasking problem. Evolutionary multi-tasking algorithms share knowledge among individual tasks and accelerate the convergence of multiple optimization tasks (Liang et al., 2019).

Relevant domains to multi-task are transfer learning and multi-objective optimization. A linearized domain adaptation (LDA) strategy transforms the search space of a simple task to the search space similar to its constitutive complex task (Bali et al., 2017). Researchers explored the use of transfer learning to tackle the problem of dynamic multi-objective optimization (Jiang et al., 2018). This method can significantly speed up the evolutionary process by reusing past experience and generating an effective initial population pool. The formulation of multi-objective optimization allows us to share the underlying similarity between different optimization exercises and automates the information transfer, which improves the convergence (Gupta et al., 2016).

Inspired by the methods mentioned above, our method solves a multi-tasking problem by effectively taking advantage of the information from a few building blocks. Our method directly applies to real-world scenarios to identify potentially-infected subjects. So far, we found that this problem is under-explored.

## 3. Methodology

**Figure 1:**
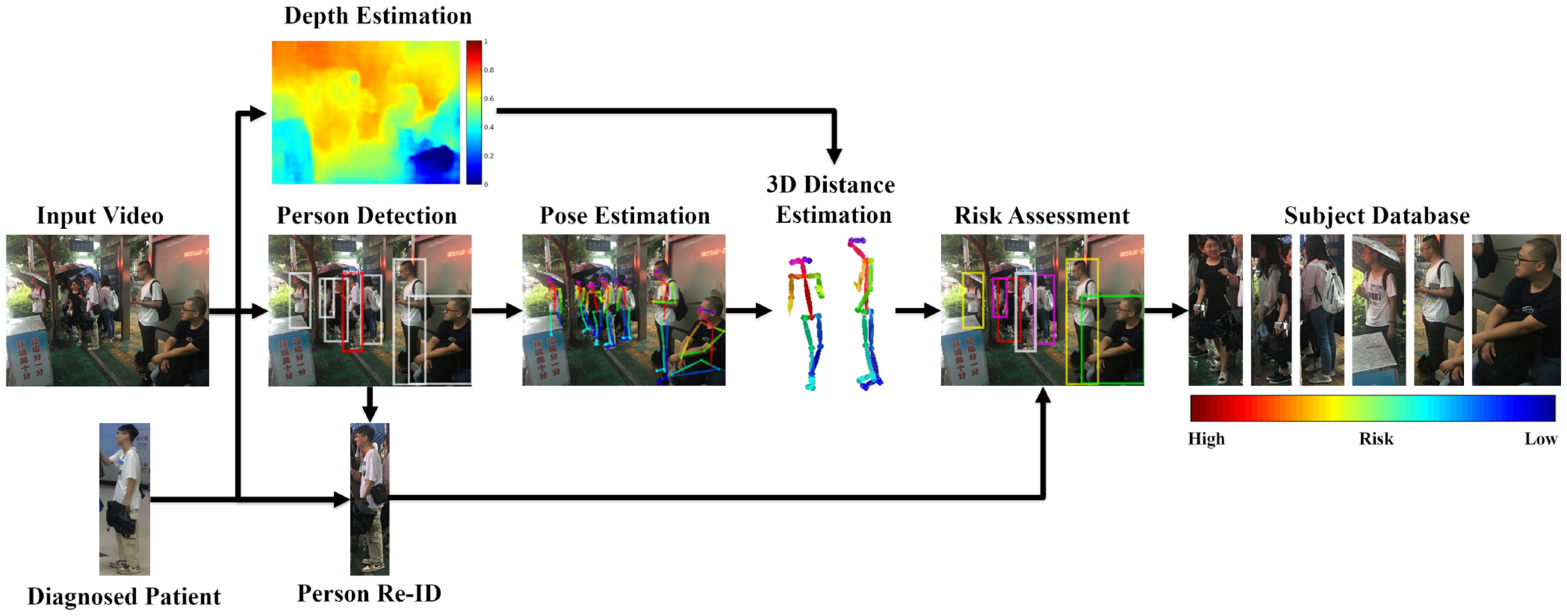
Pipeline of our method. Written informed consent for the publication of this image was obtained from the identifiable persons.

The key contribution of our method is to quantitatively model the principle of Close Proximity Interaction (Salathé et al., 2010), based on the state-of-the-art techniques in computer vision. The input to our workflow is video sequences **VS**_*i*_, *i* = 1, 2, 3, ⋯ , *N*_*c*_, captured by multiple (*N*_*c*_) cameras. These cameras are potentially non-overlapping and installed at different locations. The search starts with a diagnosed patient P^*^, who is confirmed in the clinic with the pandemic disease. The goal of this work is to identify the contact graph (**CG**) and quantitatively evaluate their potential infection risk (**PR**) with the principle of close proximity interaction. The workflow of our method is presented in Figure 1.

Our method successfully evaluates the infection risk and requires to solve multiple problems simultaneously. The tasks range from the fundamental problem to extract human from an image, to identify the same subject across different cameras and eventually to evaluate the infection risk for potential subjects. The knowledge learned from one task is harnessed for use in other tasks. The final goal of infection assessment can only be achieved by integrating the knowledge from prior sub-tasks. We describe our method as two main stages: (1) identifying the potentially-infected group, (2) modeling close proximity interaction.

### 3.1. Identifying the Contact Graph

The first step of our method is to identify the potentially-infected group of subjects. This includes a couple of sub-tasks: (1) segmenting the persons from the image, (2) re-identifying the diagnosed patient P^*^ across different cameras, (3) constructing the contact graph (**CG**) by adding the individuals who appear in the same image with the patient P^*^.

Faster R-CNN (Ren et al., 2015) is used for person segmentation as the first step of our method. Faster R-CNN extends Fast R-CNN by unifying the Region Proposal Networks (RPNs) with the original network architecture to break the bottleneck of computing time cost. RPNs are a kind of fully-convolutional network (FCN) for generating detection proposals, sharing convolutional layers with Fast R-CNN. RPNs and Fast R-CNN are trained independently. The unified architecture provides convolutional features for both object detection and region proposal tasks.

We leverage an open-source project, SVDNet (Sun et al., 2017), for person re-identification. We choose this method because of its mesrits in computational performance and comparable accuracy as the state-of-the-art. This work optimizes the deep representation learning process with Singular Vector Decomposition (SVD). It is motivated by the observation that after training a convolutional neural network (CNN) for classification, the weight vectors within a fully-connected layer (FC) are usually highly correlated.

We use a graph representation to model the contact network. Each edge **E** is a sequence involving two subjects *S*_*A*_, *S*_*B*_ as the graph nodes. Two nodes can be connected with multiple edges since two subjects can encounter each other at multiple locations.

### 3.2. Modeling Close Proximity Interaction

We model the principle of close proximity interaction by extracting contextual knowledge from the surveillance videos. The knowledge includes personal identity (acquired from the previous stage), spatial and temporal information. The latter two components describe the movement trajectories of individual persons in the 3D space. These are used o evaluate the extent of interaction proximity among subjects in the contact graph (**CG**). This is based on the assumption that the infection transmitted via droplet is critically related to the physical distance between individuals.

For each edge **E** in the contact graph **CG**, we segment the sequences from the video containing both subjects *S*_*A*_, *S*_*B*_ on the edge **E**. For each sequence, we perform three tasks: (1) depth estimation, (2) posture estimation, and (3) risk evaluation.

For the task of depth estimation, we use the existing method (Zhou et al., 2017). This method estimates the depth information from unstructured video sequences captured by a monocular camera. The acquired depth information is used to estimate the joint trajectories in the 3D world robustly.

For the task of posture estimation, we use OpenPose (Cao et al., 2017), an open-source real-time multi-person pose estimation system. We use the provided body and hands detector to obtain the 24 key points of each individual in the image. Two-dimensional position information can be acquired by the pre-trained model.

Third, we calculate the Euclidean distances between all visible keypoints of two people separately and seek the joint on the identified patient with the smallest distance to a potential subject. The distance of joints in the 3D world can be computed with the pose positions on a 2D image and the extracted depth information. We compute the infection risk as:

(1) $$\begin{array}{l} R=\frac{1}{N_{j}}\overset{N_{j}}{\underset{i\;=\;1}{\sum }}D\left(J_{i},J_{m}^{*}\right) \end{array}$$

(2) $$\begin{array}{l} J_{m}^{*}=\underset{j}{\arg\;\min\;}D(J_{i},J_{j}^{*}),i,j=1,\cdots ,N_{j} \end{array}$$

where *N*_*j*_ is the number of joints. $J_{m}^{*}$ indicates the joint on the identified patient with the minimum distance to the potentially-infected subject. $D\left(J_{i},J_{m}^{*}\right)$ computes the distance between the *J*_*i*_ joint on the potentially-infected subject and $J_{m}^{*}$ on the patient. The risk *R* indicates the average distance of all joints on the potentially-infected subject to $J_{m}^{*}$ on the patient. We iterate this process for all identifiable subjects in the image.

## 4. Results

### 4.1. Hardware and Software

Our algorithm runs on a standard PC (CPU: Intel i7 9700, GPU: RTX1080Ti, RAM:16G). The algorithm is implemented in the Python environment. The deep learning models are implemented with the open-source framework, TensorFlow.

### 4.2. Person Detection

The model is trained on COCO dataset for 160k iterations, starting from a learning rate of 0.02 and reducing it by 10 at 60k and 80k iterations. In RPN network, we use 5 scales with box area of the square of 32, 64, 128, 256 and 512 pixels for anchors and 3 ratios of 0.5, 1, 2. There are 256 anchors per image to use for training in total. The Faster R-CNN outputs the individual detection results. The average time cost for this task is 0.011 s.

### 4.3. Person Re-identification

**Figure 2:**
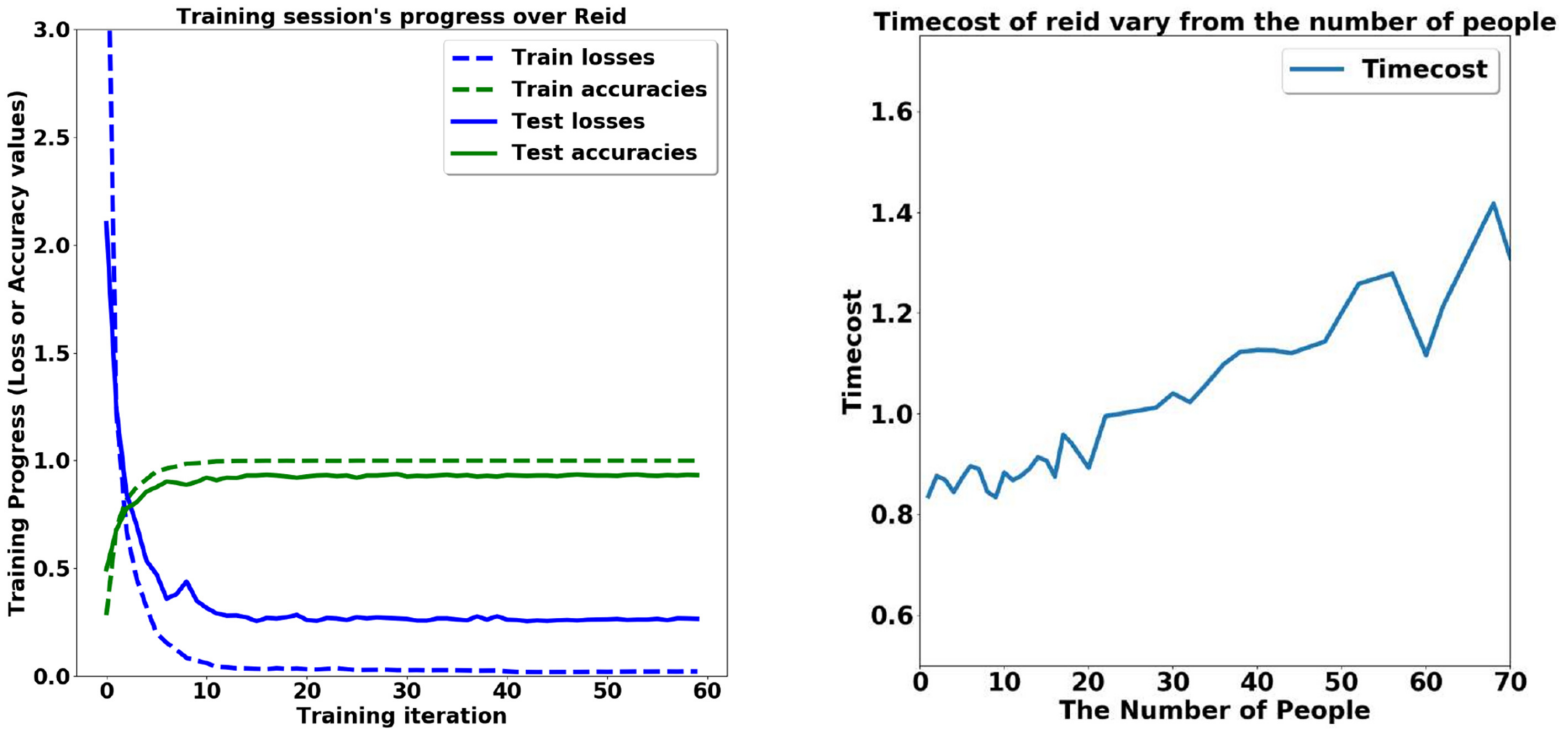
Time performance in the task of person re-identification. **(Left)** The training stage. **(Right)** The time cost given different number of persons.

We use the database of CASIA (Yu et al., 2009; Chen et al., 2017c) to train our network model for the task of person re-identification. The task of person re-identification achieved 88.24% top-1 accuracy, mAP = 70.68% only with softmax loss. The training strategy of combining Part-based Convolutional Baseline (PCB) and ResNet50 achieves state-of-the-art performance. We use Adam Optimizer with the learning rate of 0.1, the batch size of 32, and the stride of 2. Dropout strategy is adopted to avoid the over-fitting problem, and the drop rate is set to be 0.5. The process of the training is presented in Figure 2.

**Figure 3:**
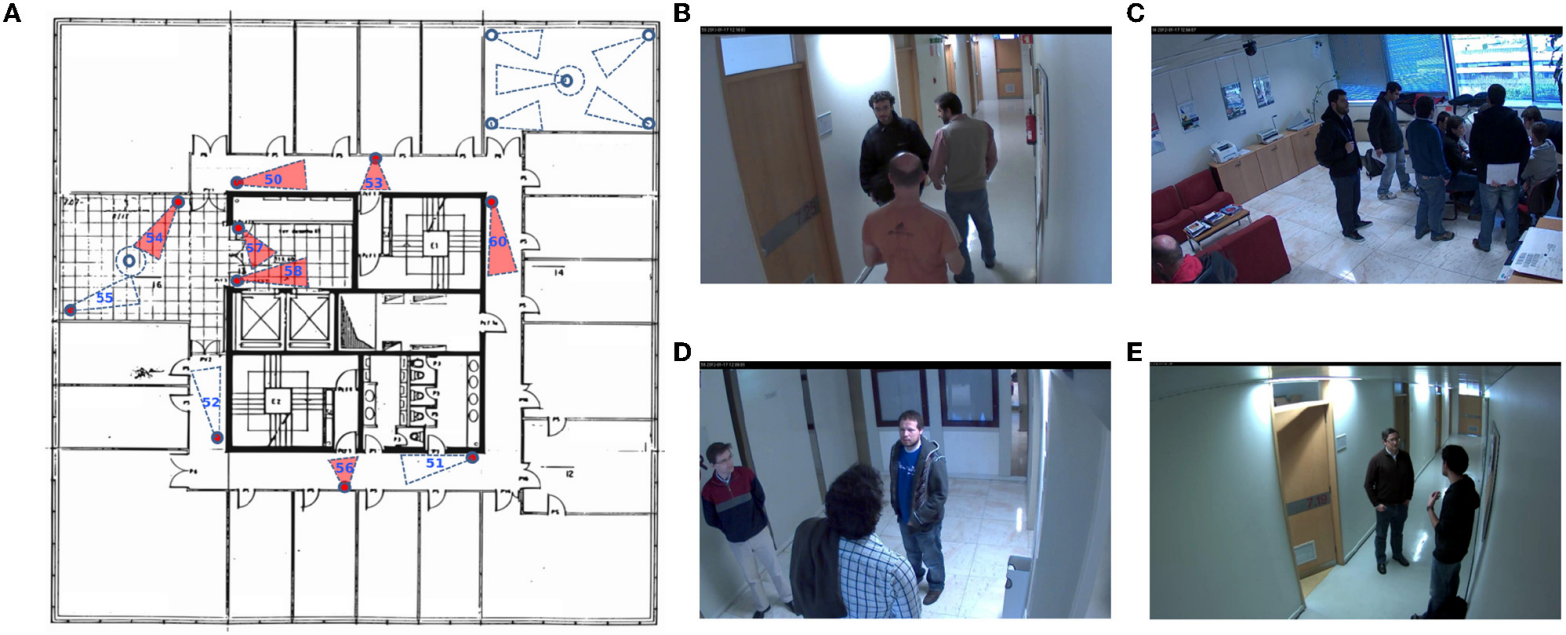
Illustration of public HDA dataset (Nambiar et al., 2014). **(A)** Camera layout; **(B)** Camera 50; **(C)** Camera 54; **(D)** Camera 58; **(E)** Camera 60.

The number of people in the image critically affects the computation load of our method. The initial process for person segmentation leveraging the Faster R-CNN is insensitive to the number of people. The average time cost of one single image is 0.8 s. However, the amount of time spent on subsequent steps is affected by the number of people involved. The person re-identification method takes segmented individuals as input and seeks the target person among these people. The increase in the number of people leads to greater time consumption, increasing from 0.8 s of 5 persons to 1.4 s of 70 persons (shown in Figure 3). The time cost of multi-person pose estimation based on OpenPose is 0.8 s for 4,032 × 3, 024 pixels' image. Thus, the total time cost of our method is no more than 3 s, far below the average time required by labor.

### 4.4. Experiment on Public Dataset

We here evaluate our method on a public dataset, HDA (Nambiar et al., 2014). We choose this dataset because they offer the video sequences in an uncropped way so that the depth information can be obtained. The HDA dataset is originally constructed for person re-identification, with 18 cameras recorded simultaneously during 30 min in a typical indoor office scenario at a busy hour (lunchtime) involving more than 80 persons. The cameras are located on three floors, and 13 cameras have been fully labeled. The floor plans are offered on the dataset website^1^. To accurately evaluate our method, we choose four labeled cameras (camera ID: 50/54/58/60) on Floor 7 and analyze the contact patterns between the detected persons. Camera 50 and 60 are placed toward the corridor, Camera 54 captures an indoor office room, and Camera 58 monitors a lobby at the lift. These are typical scenarios in an office environment.

**Figure 4:**
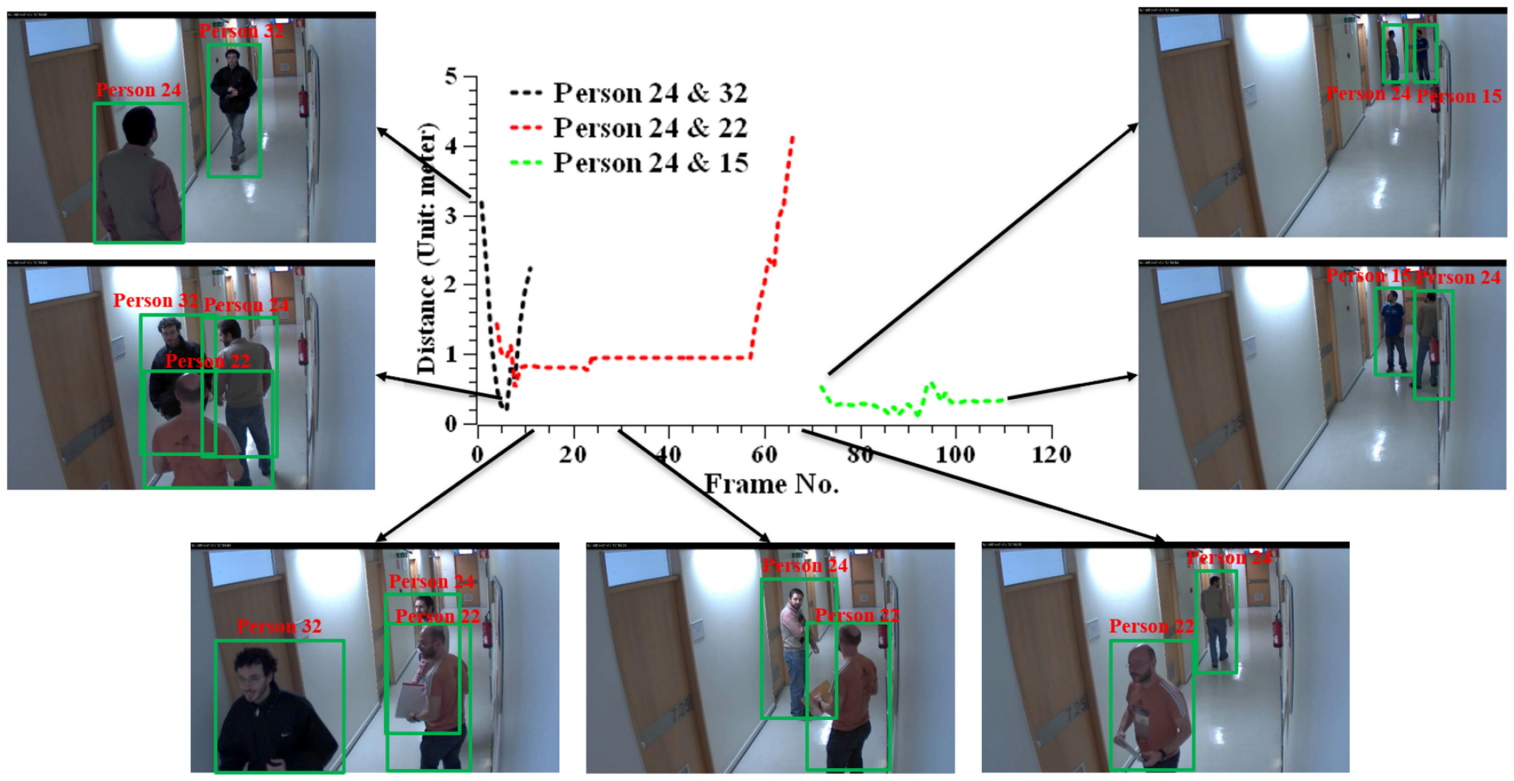
Measuring the distance between subjects in Camera 50 from the public HDA dataset (Nambiar et al., 2014).

Figure 4 plots the distance between subjects (marked as ID: 15, 22, 24, 32) in Camera 50. We here assume that the subject of ID:24 is the diagnosed patient and compute the relative distance with other subjects who appear in this camera. Because there is no direct body contact in this scenario, we use the distance between the body centers (the hip joints) of two subjects for the visual demonstration. The results show that the predicted distance between the two subjects is consistent with the perception in the real world. It shows that our method can reliably capture the interaction within close proximity.

### 4.5. Multiple Scenarios

To further verify our method, we consider public places with a massive flow of people where the infectious disease spreads quickly. Three typical scenarios are considered here: a bus station, a bus compartment, and a hospital.

#### 4.5.1. Bus Station

**Figure 5:**
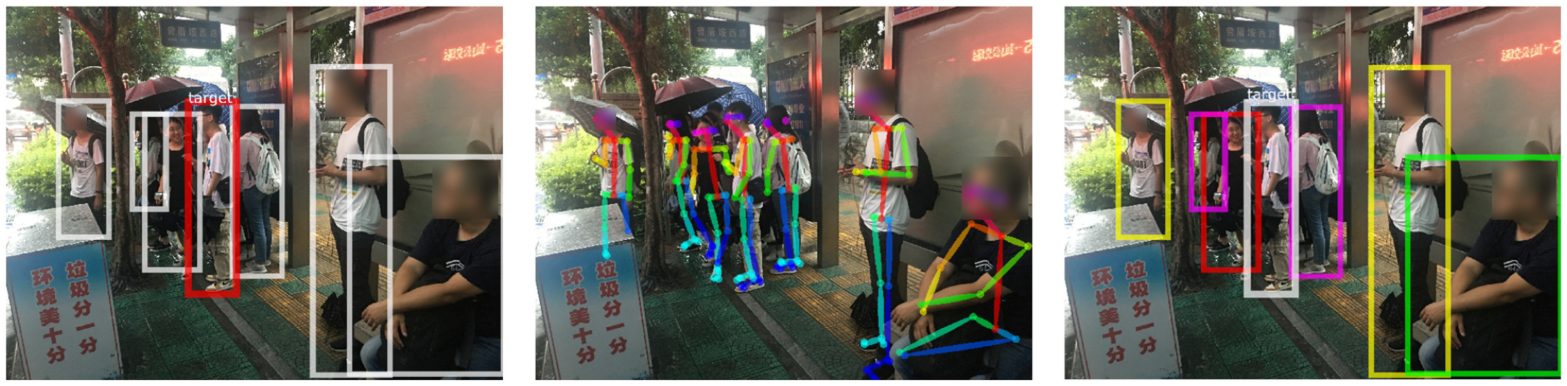
Experimental results at a bus station. From left to right: the detected persons, the pose estimation and the ranking order of infection risk.Written informed consent for the publication of this image was obtained from the identifiable persons.

The scene is rainy and the background is chaotic (Figure 5). Many people are partially shielded by umbrellas. In the middle of the image, the crowd is so dense that only the heads can be seen. Another point worth noting is that the distance between the person and the camera varies greatly. Thus, the relative size of the skeleton varies greatly, which is prone to influence the results of risk ranking. However, through the robust method combining depth and posture estimation, risk ranking results are satisfactory.

#### 4.5.2. Bus Compartment

**Figure 6:**
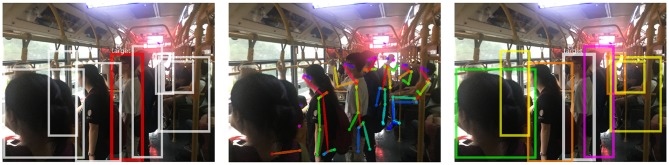
Experimental results in a bus compartment. From left to right: the detected persons, the pose estimation and the ranking order of infection risk. Written informed consent for the publication of this image was obtained from the identifiable persons.

Insufficient light in the bus compartment makes it harder to achieve the person retrieval (Figure 6). Besides, the target person is photographed from a side view rather than the same angle as his identity photo. Different perspectives are also an important factor causing difficulties in person retrieval. Results show that our method is robust to the view variations.

#### 4.5.3. Hospital

**Figure 7:**
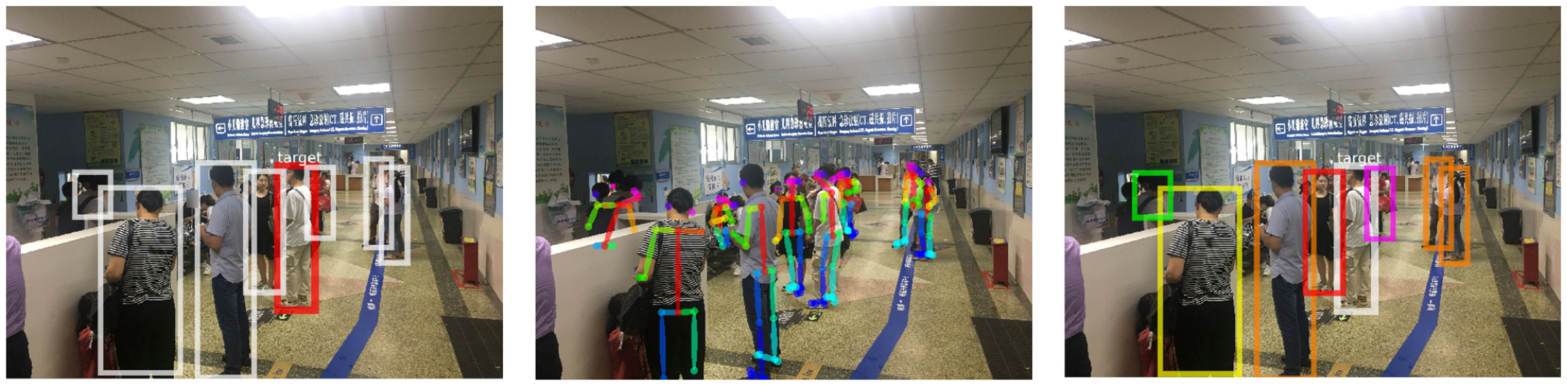
Experimental results at a hospital.From left to right: the detected persons, the pose estimation and the ranking order of infection risk. Written informed consent for the publication of this image was obtained from the identifiable persons.

The mutual occlusion between individuals is significant in this case (Figure 7). Considering the pose information we use is two dimensional, it is difficult to determine the exact distance between people. With the depth information, infection risks we obtained are consistent with our visual, intuitive judgment.

### 4.6. Comparison With User Study

To evaluate the reasonableness of our method in assessing the risk of infection, we used the risk assessment obtained by human subjects as a comparison baseline.

#### 4.6.1. Participants

Ten volunteers (5 males and 5 females) with an average age of 21 and SD of 3.5 were recruited in this study. They are all undergraduate and graduate students in the department of information science. Written agreement to participate in this study was obtained from individual participant after explanations of this study. They all agreed to join this study for free.

#### 4.6.2. Procedures

Participants were invited to the lab and conducted this experiment. After explaining the task details, they signed the agreement of participation. They were instructed to rank the infection risk of all detected persons in each video, given the diagnosed subject. They were not aware of the purpose of this study, as the comparison baseline of our proposed algorithm.

We used all three scenarios (bus station, bus compartment, and hospital) in the previous section. Participants were presented with a short sequence of videos, They were instructed to sort the infection risk of all detected individuals in the image based on common sense or intuition. Starting from the candidate with the highest perceived risk, they associated with the candidate with the rank number from 1 to N (N is the number of candidates in each image). No judging criteria were given. We started the timer when the participant sees the image and started marking it without explicitly informing the user of timekeeping. Interviews were conducted after participants finished the previous procedure by asking open questions and collecting their subjective feedback on how they perceived and ranked the risk. Each participant spent around 20 min to complete the study.

#### 4.6.3. Quantitative Findings

**Figure 8:**
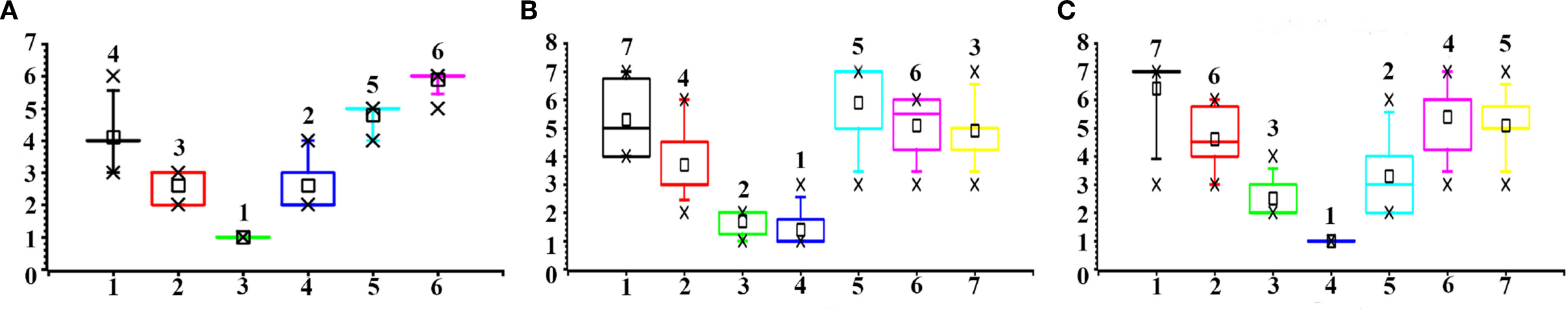
Comparison of manual ranking and our method. Horizontal axis: the candidate ID in the image (from left to right). Vertical axis: the ranking order. A smaller value of the ranking order indicates a higher risk of infection. **(A)** Bus station; **(B)** Bus compartment; **(C)** Hospital.

We compared the ranking result from our method and the user experiments (Figure 8). The bar plots show the distribution of the ranking order, while the number on top of each box is the proposed order by our method. The results show that the ranking order of infection risk is consistent between our method and human subjects. Participants achieved a higher degree of consensus with the highest and lowest ranking candidates. For the examples of both the bus station and the hospital, all participants identified the person (ID = 3 and ID = 4 in these two respective examples) closest to the diagnosed patient as the top candidate of infection risk. For the example of the bus compartment, the choice of the top candidate is distributed to two options (ID = 3 and ID = 4). However, for other options between the highest and lowest ranking candidate, participants showed a higher degree of variation.

In terms of time cost, our method requires far less time (3 s) to process one image, than the time cost required by our participants (2 min). During the decision flow, when the participants ranked the risk order, we observed that it requires significantly less time to identify the person of the highest risk than the rest choices. This is consistent with the high degree of consensus in the candidate selection. We believe this shows the advantages of our method in accuracy and efficiency. The reasoning behind the decision process of human participants is to be explained in the following paragraph.

#### 4.6.4. Qualitative Findings

We interviewed the participants and collected their feedback and comments. We asked about how they decided the ranking order, and all participants mentioned the factor of the distance between the candidate and the diagnosed patient. This confirms the principle of close proximity interaction. Six participants explicitly pointed out that the fact that the top candidate is conversing with the diagnosed patient in the examples of the bus station and hospital critically shapes their decision. This is also consistent with the transmission route of the droplet. When people are having a face-to-face conversation, the droplets are more likely to spread out to the person in the conversational group. The carried virus in the droplet causes the infection. For the ranking decision with lower possibility, participants agreed that it is more difficult to decide since more than one candidate is located at a similar distance with the diagnosed patient. However, they also mentioned that since the rest of the candidates are not exposed to the high infection risk, their significance to infection control deserves less attention.

## 5. Discussion

In this section, we discuss the insights we learned from our experience, in particular typical failure cases and limitations in our experiments.

### 5.1. Failure Case Analysis

The building blocks critically determine the success of inferring the close proximity interaction in the upstream workflow. Here we identify the failure cases caused by two components: person re-identification and distance estimation.

**Figure 9:**
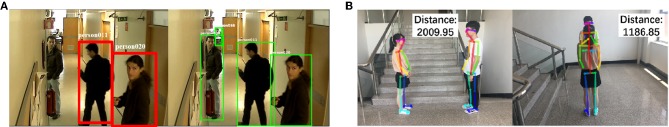
Failure cases of our method. Images in **(A)** are from the public dataset HDA (Nambiar et al., 2014). Written informed consent for the publication of image **(B)** was obtained from the identifiable persons.

The state-of-the-art methods in person re-identification still face significant challenges in a complicated environment. The current accuracy of re-identification in our method is 88%. In selected scenarios, the method in our work fails to identify the same person in two different camera views. This is caused by the relative perspective between the person in the view and the camera perspective. Improving the method of re-identification is the solution to this problem. Figure 9A presents one typical failure case. The person on the left of the image is about to exit from the corridor and partially occluded. This creates a detection failure.

Reliable reconstruction of 3D information from the 2D image is still an open question in the domain of computer vision. Although we propose an efficient method to infer the depth information and integrate with the 2D posture, failure cases still arise due to occlusion and viewpoint perspective. For the former case, if the two persons are standing in line with the camera (shown in the right image of Figure 9B), the detected key points will be almost mixed together. At this time, it is significantly challenging to predict the distance between the subjects.

## 6. Limitations

First, only direct infection is considered, while the indirect infection is neglected. Some bacteria or viruses will remain on objects such as escalator rail, doorknob, shopping cart, etc. handled by infected patients. Though their infection may be weakened to varying degrees, it still poses potential threats to indirect infection. We did not take these contaminated objects into account yet. Object detection and tracking techniques will help to locate these objects. It is challenging to accurately determine whether a person is in direct contact with an object rather than just being close to it due to the factors of occlusion and overlapping. When the contaminated object is sheltered from persons or multiple objects overlap each other, the visible part of the object is insufficient to provide sufficient information for making a judgment.

Second, formulating the infection risk assessment criteria based on vision-level rather than chemical analysis also presents a unique set of challenges. Obtaining the exact distance between people in practical circumstances is necessary for verifying the estimated distance by our method. Besides, the potential risk of infection varies according to different environmental set-ups and transmission routes of infectious diseases. A confined space like a room may lead to a higher risk than an open space. The cumulative effect of continuously contact over a while rather than a particular moment is difficult to measure. Besides, it is worth pointing out that we do not take the intra-person variations of immunity into account since it cannot be measured at the vision-level.

## 7. Conclusion

This paper proposes a novel method to represent the potentially-infected group of people as a graph structure. We also model the principle of close proximity interaction by robustly analyzing the physical distance between subjects in the 3D world. This vision-based approach can re-identify diagnosed patients with infectious diseases and evaluate the infection risk of people who have contacted them. We evaluated our method in various scenarios, including indoor office, bus station, bus compartment, hospital. The comparison with the process of manual analysis shows that our method achieves consistent results but significantly reduces the time cost.

There are a few directions for our future work. Our current work focuses on the direct contact between the subjects and neglects the indirect contact between subjects via objects. It is highly likely that the objects in close contact with the diagnosed subject contain the virus and thus lead to disease spread. Investigating the indirect infection caused by contaminated objects is in line with our future work. Besides, deploying our method in an in-the-wild study could validate the effectiveness of our method in the real world. One potential scenario is to predict the absentee statistics of the childcare center, given the surveillance camera videos. This could offer advice to parents and administrators concerning the status of the disease infection on both individual and group levels.

## Data Availability Statement

Publicly available datasets were analyzed in this study. This data can be found here: http://vislab.isr.ist.utl.pt/hda-dataset/.

## Ethics Statement

The studies involving human participants were reviewed and approved by School of Informatics, Xiamen University. The patients/participants provided their written informed consent to participate in this study.

## Author Contributions

SG conceived, designed the analysis, and wrote the majority of this manuscript. MJ and XG conceived, designed the analysis, and revised the manuscript. XS conceived the analysis and participated in the writing of the manuscript. JY and HW collected and performed the analysis. FX read and revised the manuscript.

### Conflict of Interest

The authors declare that the research was conducted in the absence of any commercial or financial relationships that could be construed as a potential conflict of interest.

## Footnotes

^1^Available online at: http://vislab.isr.ist.utl.pt/hda-dataset/.
